# Preparations for and practices of online education during the Covid-19 pandemic: A study of Bangladesh and Nepal

**DOI:** 10.1007/s10639-021-10659-0

**Published:** 2021-07-28

**Authors:** Sagun Shrestha, Saifa Haque, Saraswati Dawadi, Ram Ashish Giri

**Affiliations:** 1 School of Applied Language and Intercultural Studies (SALIS), Dublin City University, Dublin, Ireland; 2 Department of English, East West University, Dhaka, Bangladesh; 3 Institute of Educational Technology, The Open University, Milton Keynes, UK; 4 English Language Centre, Monash University, Melbourne, Australia

**Keywords:** Higher education, Covid-19, Online education, Challenges, Mental wellbeing

## Abstract

Online education has been adopted widely to address the educational chaos created by the Covid-19 pandemic. Reports on its constraints and challenges appear daily in the global media. However, accounts of teachers’ and students’ experiences of this abrupt shift in pedagogical modality are conspicuously absent in the available literature. This article reports the findings of a study that explored teachers’ and students’ experiences of online education during the pandemic in the context of higher education in Bangladesh and Nepal. The online survey with 147 students and 76 teachers and interviews with a sub-sample of 17 participants indicate that they adapt the action potentials of the digital artifacts to local contexts and use them in the best possible ways to facilitate their communication and enhance student learning in difficult circumstances. The major challenges and constraints they experience in transitioning to online education include poor network, lack of digital skills, lack of technological support from institutions among others. The study findings indicate some pressing policy, pedagogical and research implications, which are discussed in the final section.

## Introduction

The pandemic outbreak of Covid-19 has created serious disruptions in educational activities. Delivering learning to homes has been challenging to teachers in most under-resourced contexts, where the accessibility, availability, and use of technology in education are not widespread (Khan et al., 2012). Apart from this, teachers’ poor digital skills prevent them from delivering effective learning (Laudari & Maher, 2019), and little thought has been given to train them on how to manage students' social, physiological, and psychological issues. While higher education institutions have generally managed to implement teaching and learning activities using online tools globally, the systems and resources in developing countries such as Bangladesh and Nepal are inadequate (Dawadi et al., 2020; Uddin, 2020). University teachers in developing countries often resort to using available technological resources to cope with the challenges and to improve their teaching and learning (Shah et al., 2020). Consequently, the transition to online teaching was not smoother in developing countries compared to developed countries (Saeed, 2020).

This article, which is based on a binational study of English as a Foreign Language (EFL) teachers and students of higher education institutions in Bangladesh and Nepal, explores how teachers and students prepare themselves for handling the pedagogical shift, what their experiences are in using the digital devices/artifacts for managing teaching and learning, what challenges they encounter, and how the limitations impact their teaching and learning. The following section introduces the broader research context—digital technology in higher education settings.

### Digital technology in higher education: The broader research context

Using ICT in higher education has been increasingly common since the advent of the Internet, though the degree of its use differs from one context to another. A plethora of research claims that digital technology meets not only the changing need of higher education students, but it also enhances learning (Alzahrani & Seth, 2021; Becker, 2017; Du Toit & Verhoef, 2018; Lai, 2011; Laudari & Maher, 2019; Underwood, 2009; Waghid & Waghid, 2016). Lai (2011) and Underwood (2009), for example, assert that technology is used in all higher education institutions, though to varying degrees, to support traditional forms of teaching. Similarly, Becker (2017) and Waghid and Waghid (2016) affirm that flexi-learning, e-learning, and blended-learning have now become a norm and alternative to face-to-face teaching and learning in higher education. Additionally, discussing its usefulness in tertiary education, Neupane (2017) states that technology helps create a community of practice where learners of English, through interactions and collaborations, acquire language proficiency, advance critical and problem-solving skills, increase their engagement and retention, and obtain higher academic achievement. Digital learning, therefore, has been a common phenomenon in higher education as many higher education institutions today are using online and/or blended learning in many of their course offerings.

Although digital technology, such as the use of digital artifacts, web resources and platforms, has been in practice for some time now, its use in and impact on learning in higher education has largely remained unexplored. According to Lai (2011), little is known about how university teachers use digital technology in teaching and learning, and how it is embedded in pedagogy, along with its potential impact on students. Mohammadi et al. (2021), in their study in Afghanistan found out that lack of policy, guidelines, and detailed policy documents was one of the key challenges for implementing digital technologies such as the learning management system (LMS) in higher education; however, Alzahrni and Seth (2021) argue that learners’ expectations of positive advantages from using technologies (personal outcome expectations) have a significant influence on their continuous intention to use LMS. The technologies that help to provide long-distance courses, such as the learning management systems, "provide an interactive learning environment and automate the administration, organization, delivery, and reporting of educational content and learner outcomes" (Turnbull et al., 2019, p. 1). Laudari and Maher (2019), in a study at a teacher training institution (faculty) in a developing context, conclude that there are two types of barriers that mitigate against effective use of digital technology in teacher education. First, it faces first-order (external) barriers, such as lack of resources and training, unconducive policy and administration, rigid curriculum and assessment, and second-order (internal) barriers which relate to teachers' own beliefs, motivation, and attitude towards technology (Ertmer, 1999). With the affordable availability of technology and its increasing use, the first-order barriers to technology are less noticeable; however, second-order barriers are becoming more influential. Shah et al. (2020) in their study found that university teachers in Pakistan use less expensive and widely available options such as Facebook/Google groups as alternative arrangements as institutional learning management systems are absent.

To sum up, in higher educational institutes, technology can be used in both synchronous and asynchronous ways. Teachers use technology not only to prepare and distribute course materials but also to communicate with students. Besides, technology is used for academic research and administrative activities (Rumanyika & Galan, 2015). Using technology in higher education gives benefits in many ways, for example, technology has the potential to erase the barriers to education created by space and time, and it can also provide access to lifelong learning (Baldwin, n. d.). However, in resource-constrained contexts, using technology in higher education is still a challenge (Stantchev et al., 2014).

### Digital learning: A new field of inquiry

The issue of if, and to what extent, digital/mobile technologies have impacted the culture of practices, in particular, education and communication practices, has been widely researched and well-documented (Viberg &  Grönlund, 2013). A distinct specialised field of inquiry, known as digital learning (*dLearning*), thus, has emerged which is attracting the attention of educators, assessors and researchers globally. Digital learning encompasses the application of a wide spectrum of learning practices including blended and virtual learning (Davis, 2020). Several studies claim benefits of digital technology in terms of its contribution to (a) knowledge construction (Chang & Hsu, 2011; Cheng et al., 2010; Hsu, 2012); (b) enhancement of learning (Abdous et al., 2012; Sandberg et al., 2011; Stockwell, 2008); and (c) providing novel, creative and entertaining learning contexts (Song & Fox, 2008). Furthermore, digital technology has radically transformed the teaching and learning of English as a second/foreign language (ESL/EFL) which reportedly assist teachers to personalise their teaching (Oberg & Daniels, 2013), facilitate group work and create collaborations among learners (Pemberton et al., 2010), enhance learner autonomy (Murphy et al., 2012), increase engagement in learning tasks and in creating problem-solving tasks (Cook, 2010; Driver, 2012), and assist teachers to provide instant feedback (Voelkel, 2013).

The pandemic, however, has exposed constraints and challenges that teachers and their students experience in using digital technology in education. While higher education institutions are investing to enhance the infrastructure, there are arguments and counter-arguments about whether it is feasible and appropriate to equitably do so (Rahman, 2020). Several studies (Khan et al., 2012; Laudari & Maher, 2019; Rana, 2018; Salehi & Salehi, 2012; Shrestha, 2016) have identified several barriers to *dlearning* in developing contexts including lack of support, limited ICT infrastructure, insufficient funds and lack of proper plan to integrate technology in education. These barriers can be classified as school-level barriers. Other barriers are teacher-level barriers such as teachers' lack of knowledge, skills and time, and system-level barriers such as policy level weaknesses affecting the integration of technology in education (Balanskat et al., 2006). These barriers are, as discussed below, challenges in adopting technology in teaching and learning, especially during the transition to online education.

### Transitioning to online education during the Covid-19 pandemic in Bangladesh and Nepal

The education system is facing a crisis with the spread of the coronavirus, with over 190 countries having gone through some form of school closures that has impacted more than 7 billion students (The World Bank, 2020). The majority of governments directed academic institutions to stop their regular instruction and switch it to online teaching (Daniel, 2020). Hodges et al. (2020) contend that this temporary shift of instructional delivery, which is an alternative delivery mode due to crises, is Emergency Remote teaching (ERT). Daniel (2020) emphasises that this emergency is not the time to plan for the complex institutional plans to adopt distance education, which will be implemented over months or years rather it is worth starting with what teachers know to cope with the emerging situation.

The coronavirus pandemic has massively affected education alongside people’s health (Terlecki, 2020). Governments of many developing nations, including Bangladesh and Nepal, have been unable to cope with the transition due to limited resources and inadequate infrastructure (Salmi et al., 2020). For instance, adopting online mode to manage pedagogy is not a feasible option for Bangladesh yet, even though online teaching and learning is thought to be one of the ways to combat the loss in learning (Uddin, 2020). Uddin argues that the proposed education expenditure in Bangladesh for FY21 is grossly insufficient to what is needed. Similarly, in the context of Nepal, Dawadi et al. (2020) argue that an immediate shift to online classes in the current situation is extremely difficult due to the lack of experience, stable internet access, and digital illiteracy in the parents. Despite this, e-classes in educational institutions are on the rise (Karki, 2020).

Adding to the inequitable access to education, a mental health crisis is emerging as many students have lost access to services that were offered by schools. The pandemic seems to have a negative impact on students’ and teachers’ mental well-being globally, and it is more pronounced in under-resourced contexts (Christakis & Christakis, 2020). Many students are possibly experiencing an increased level of anxiety and even depression. Teachers also have come under increased pressure not only to provide learning resources to their children or conduct lessons online but also to supervise their students' learning (Grubic et al., 2020).

The discussions above suggest that while trying to mitigate the effect of the pandemic by adapting to new modalities of delivering learning, the students and their teachers face several levels of barriers and experience (a) stress due to economic uncertainty, concern for the well-being, and anxiety about the future; (b) the challenge of returning to schools where many students have fallen behind and increased pressure to ensure catch-up with little professional development support, and (c) little access to the right technologies or the skills to use them. Despite calls for supporting teachers and students, there is little or no research on how they are supported or prepared to deal with the issues arising from the new emergencies in education in general and transitioning to online teaching and learning in particular. This study addresses the gap by investigating how teachers and students in Bangladesh and Nepal have adapted to the emergency, and what the key challenges have been in transitioning to online teaching and learning. The article also explores how higher education institutions provide support to teachers and students, meet their training needs, and help engage in a community of practice.

In particular, this study answers the following research questions: 1. What and how digital artifacts are used in higher education during the Covid-19 crisis by teachers of English/English education in Bangladesh and Nepal? 2. How do the teachers and students experience online classes and what support mechanisms are available to them to deal with psychological issues or mental wellbeing? and 3. What are the constraints and challenges faced by the teachers and students when engaging in the courses using distance mode? The digital artifacts in this paper are the digital devices and tools used by teachers, learners and institutions, and the digital resources created by them.

## Theoretical framework

**Fig. 1:**
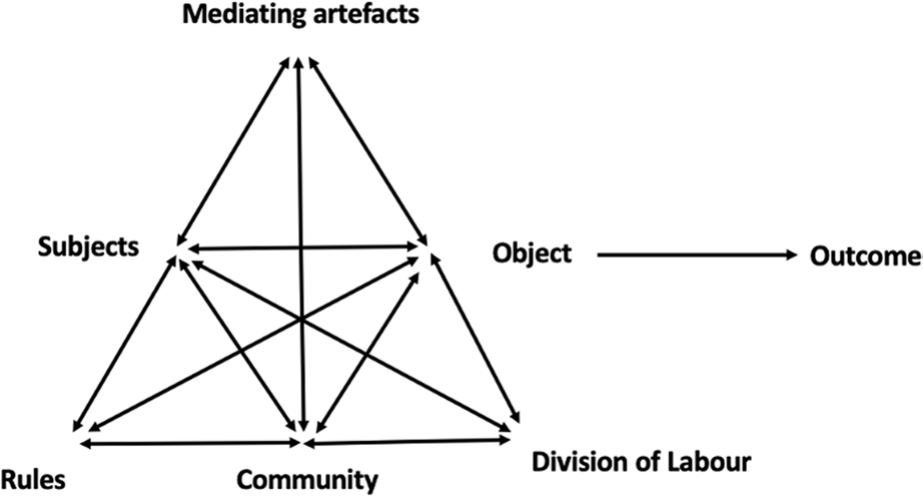
Engeström’s (1999) complex model of an activity system (adapted))

This study is guided by the cultural-historical activity theory (CHAT) that recognises learning and development as social phenomena and conceptualises human activity as object-oriented, collective and social. This theory views human relationships as interwoven with a range of contradictions which are a driving force for change. Engeström (1999) presents a triangular diagram (see Fig. 1 below) to expound on the complex model of an activity system. According to him, subjects, either individuals or groups, carry out object-oriented activity mediated by artifacts. All the components interact with rules, the formal and informal regulations that can constrain or regulate the activities, community the subject identifies and available there, and division of labour which refers to “how the tasks are shared among the community” (Yamagata-Lynch, 2010, p. 23). Engeström’s complex model of activity system helped us analyse broader activity context, the actors or subjects get involved in.

## Research methodology

This study featured a mixed-methods design comprising a survey and semi-structured interviews. A convergent parallel mixed-methods design (Creswell & Plano Clark, 2007) was employed. Therefore, both data sets were concurrently (but independently) collected; equal priority was given to both data, and the findings were combined during the data interpretation phase only.

In order to collect data, an online survey was conducted first with students and teachers, and a sub-sample of the survey participants was interviewed to explore more into the issues. The participants included tertiary level students (*n* = 147: 86 from Nepal and 61 from Bangladesh) and teachers (*n* = 76: 37 from Nepal and 39 from Bangladesh) from the faculty of English education. In the context of Nepal, around half of the teachers were from urban parts, whereas the other half were from rural areas. However, in the context of Bangladesh, almost all the teachers were from urban areas. The teachers' teaching experiences ranged from 2 to 15 years in both countries. Indeed, our population was representative in terms of gender, teaching experience, geographical location and organization types—private and public. Sub-samples of students (*n* = 4 from Nepal and 5 from Bangladesh) and teachers (*n* = 4 from Nepal and 4 from Bangladesh) were purposely selected for the interviews.

In order to ensure clarity in the instructions, the survey questionnaire and interview schedule were piloted with 2 students and 3 teachers, each with a similar background to the participants before they were released. The British Educational Research Association (BERA) ethical guidelines (2018) were followed, and informed consent was obtained from all the participants before collecting data from them. The quantitative data were analysed using SPSS version 25 and frequency distributions were calculated for most items. A thematic analysis approach, following the activity theory, was employed to analyse qualitative data, and the software NVivo R1 was used to systematically organise the data and the themes emerging through the analysis.

## Findings

The findings of the study have been organised into five major themes, which holistically describe what and how digital tools have been used, how EFL teachers and students experience online learning, what the impact on their mental wellbeing is, and what type of constraints and challenges they face during online learning.

### Mediating digital artifacts

**Fig. 2:**
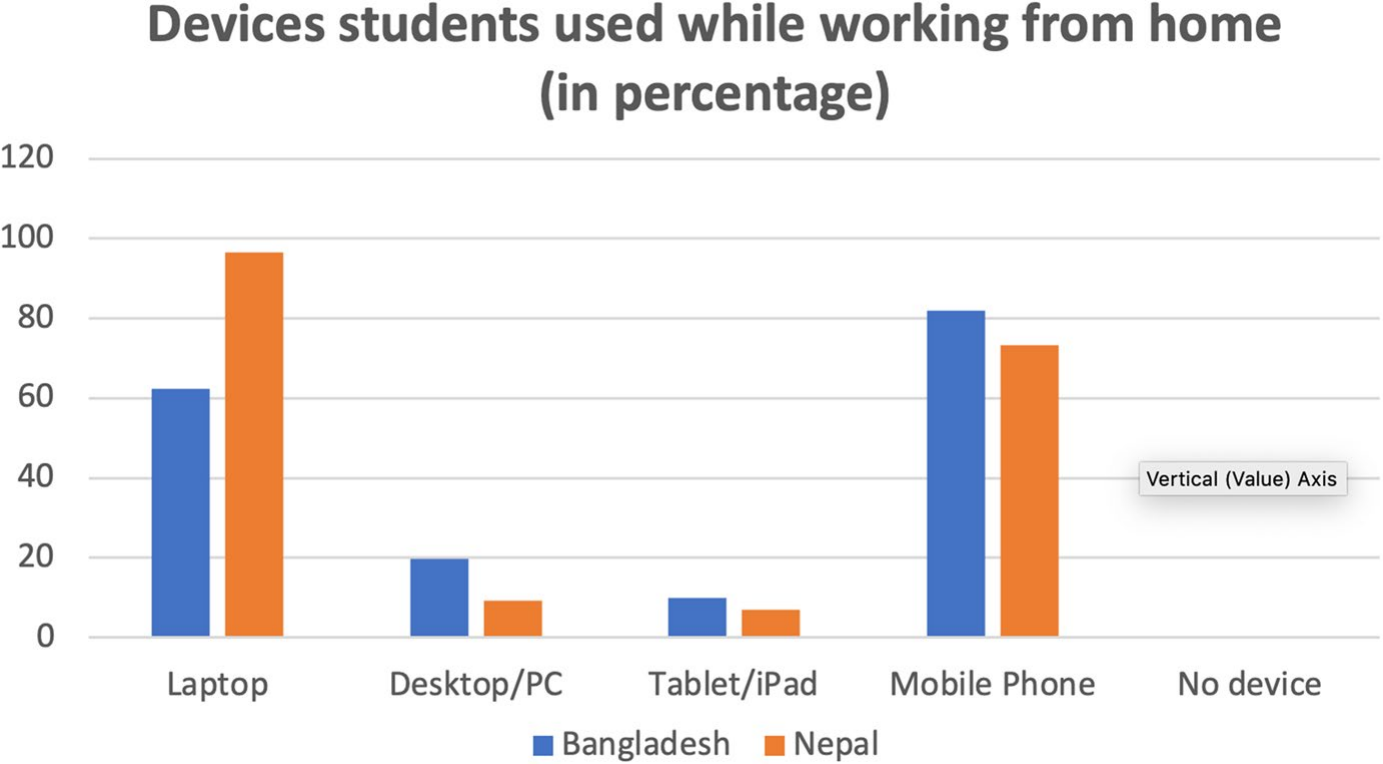
Digital devices used by students in Bangladesh and Nepal

The findings reveal that students from both countries used similar tools for their online learning. Figure 2 indicates that the majority of students in both countries used mobile phones and laptops for online learning. However, compared to Nepalese students, Bangladeshi students used mobile phones more frequently but laptops less frequently for their learning. Furthermore, tablets/iPads were the least popular among EFL students in both countries.

**Fig. 3:**
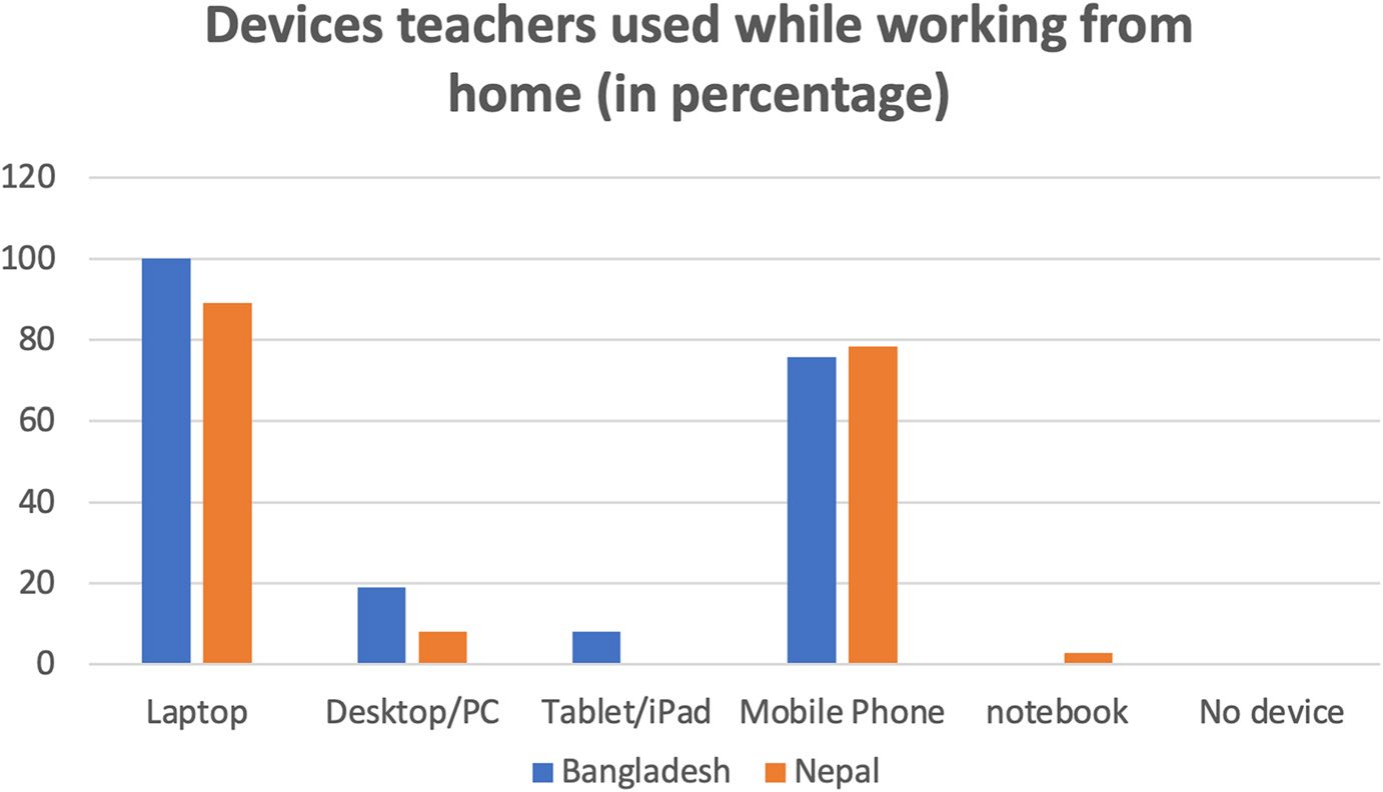
Digital devices used by teachers in Bangladesh and Nepal

Similar findings emerge through a teacher survey. Figure 3 indicates that the majority of teachers from both countries used laptops and mobile phones for their online education, but they rarely used desktop computers and tablets.

The qualitative data reveal that during their university closure, teachers in both countries contacted students through Facebook, Zoom, Google Meet, email, Messenger group, WhatsApp, Viber and phone calls. The findings further indicate that Facebook or Messenger groups were one of the most popular means of communication among EFL teachers in the target countries. In Bangladesh, WhatsApp was also frequently used by teachers to contact their students. In both countries, teachers used similar video conferencing platforms, such as Zoom and Google Meet to teach online. In Bangladesh, along with Google Meet and Zoom, teachers also used Facebook live, and some of them uploaded lecture notes on the Facebook group. In Nepal, teachers usually used email and Facebook to send important notes or reading materials to their students.

Regarding the contextual use of a digital tool, one of the students from Nepal highlighted the use of a Messenger group by saying that both the students and teachers treated a Messenger/Facebook group as their self-designed learning management system (LMS). He stated,

We have created ELT Seminar 2020, and a Facebook group of teaching practice with 28 members which we use for exchanging course-related information. Sometimes we use them to send our assignments.

It is worth noting that teachers from Nepal would disseminate information via Messenger group and students would also post their assignments on the same platform. Since they did not have any LMS, they treated the Messenger group as an important tool and used it to facilitate their communication with students and enhance students' learning. EFL students and teachers used Messenger as a tool to send and receive assignments as well. Students from Nepal reported about the creative use of this tool as follows:

The teachers use messenger and email to send assignments/questions. Messenger is easy because handwritten notes can be photographed and attached easily. One has to convert them to PDF to send by email. It looks technical (Student A).

There were also indications that some students were not able to type their answers; therefore, they had to depend on other people to submit their assignments:

While doing their assignments, they [students] would write on the notebooks, and give them to cybers for typing and submission. And they would also take the help of their friends who have laptops to type their content (Student B).

The findings also suggest that teachers used some unique strategies to promote learners' engagement while using digital artifacts. For instance, a teacher from a remote part of Nepal pointed out:

If I had to teach in the evening, I would upload the materials in the same morning. It can be in the form of a photograph or a pdf. I put leading questions in my slide and ask students to answer them before I begin my presentation. I ask students, particularly shy ones, to type their answers on chat. As there is less interaction in online classes, I do these things.

Students and teachers from Bangladesh seemed to have similar practices though they used different platforms such as Google Classroom, Moodle along with Facebook groups. A few students and teachers from a university in Bangladesh indicated they used a website called buX to facilitate online learning. buX is the online learning platform, created by BRAC University, Bangladesh, to help transition to online teaching during the pandemic.

The teacher participants in this study were also asked to report the online resources they used in the pre-pandemic situation, such as YouTube Videos, some web tools, websites, etc., and whether they had any future plans to use them. The findings presented in Table 1 indicate that most teachers did not tend to use online resources in the pre-pandemic situation. Around 8% of teachers reported that they never used online resources during the time.

**Table 1:** Teachers’ use of online resources before Covid-19. How often did you use online resources before Covid-19?

	Frequent	Percent	Valid percent	Cumulative percent
Never	6	7.9	7.9	7.9
Sometimes	28	36.8	36.8	44.7
Often	12	15.8	15.8	60.5
Very often	20	26.3	26.3	86.8
Always	10	13.2	13.2	100.0
Total	76	100.0	100.0	

However, the teachers seemed to be motivated to use online resources more frequently in the post-pandemic situation. Table 2 suggests that the majority of teachers (74%) are planning to use online resources after the pandemic. Nevertheless, around 15% of teachers reported they might not use online resources after the pandemic.

**Table 2:** Teachers’ plan to use online resources after Covid-19. Do you to plan to use any of the online resources when you return to the classroom?

	Frequent	Percent	Valid percent	Cumulative percent
No	11	14.5	14.5	14.5
Yes	56	73.7	73.7	88.2
Maybe	9	11.8	11.8	100.0
Total	76	100.0	100.0	

Similar findings have emerged through students’ data. Less than a fifth of students reported that they always used online resources for their learning in the pre-pandemic situation. The findings have been summarised in Table 3.

**Table 3:** Students’ use of online resources before Covid-19. How often did you use online resources before Covid-19?

	Frequent	Percent	Valid percent	Cumulative percent
Never	17	11.6	11.6	11.6
Sometimes	46	31.3	31.3	42.9
Often	32	21.8	21.8	64.6
Very often	27	18.4	18.4	83.0
Always	25	17.0	17.0	100.0
Total	147	100.0	100.0	

Presently, the students seem to be very much motivated to continue using online resources even in a post-pandemic context. This means the majority of students seem to have a plan to use online resources after the pandemic, as indicated in Table 4.

**Table 4:** Students’ plan to use online resources after Covid-19. Do you plan to use any of the online resources when you return to the classroom?

		Frequent	Percent	Cumulative percent
Valid	No	28	19.0	19.0
	Yes	88	59.9	78.9
	Maybe	31	21.1	100.0
	Total	147	100.0	

There are several indications that EFL teachers from both countries aim to introduce new pedagogical practices in the post-Covid situation. For instance, they reported that they might use social media to contact their students and use quiz-based tests, more online materials, blended learning courses, both online and offline activities and flipped approaches to teaching, contrasting Chugh et al. (2021)’s findings that teachers do not use social media for teaching due to a lack of awareness, skill and confidence.

### Community of practice

**Fig. 4:**
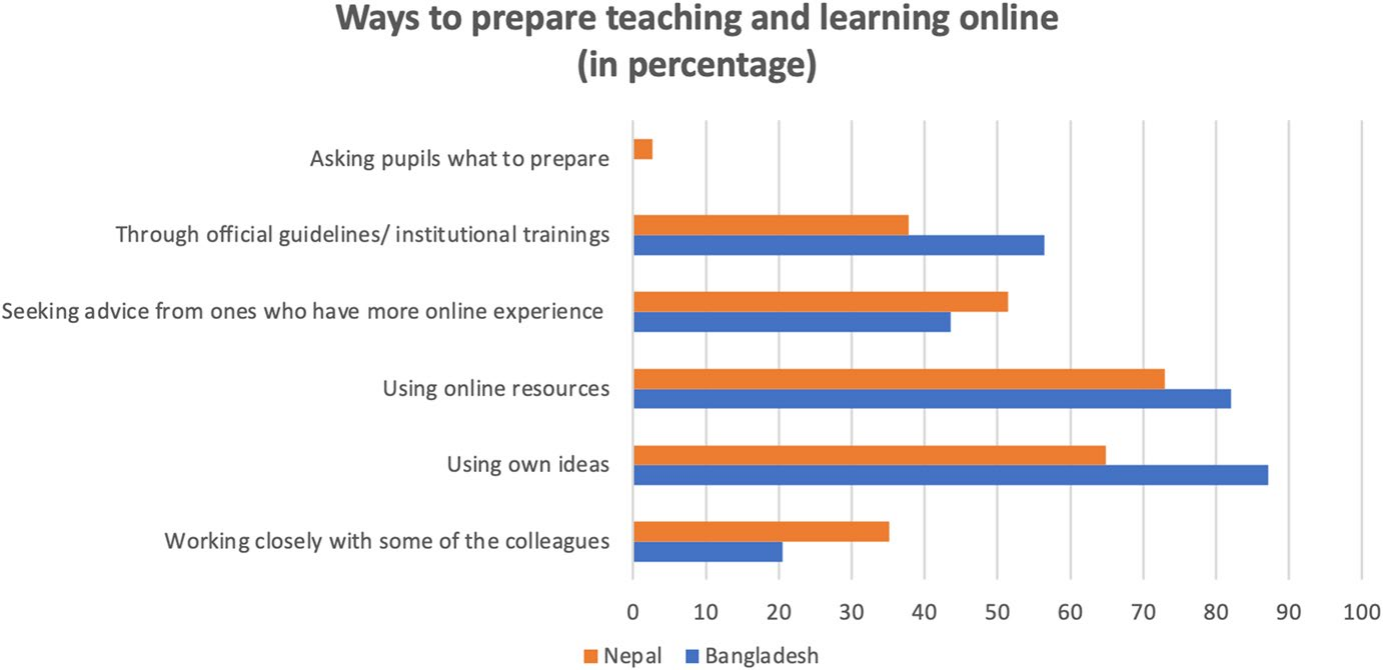
EFL teachers’ preparation strategies for online classes

The findings indicate that there was a preparation mechanism created in between teachers while transitioning to online teaching and learning. Figure 4 reveals how teachers prepare themselves for online classes.

The figure above demonstrates that EFL teachers in both countries used several strategies to prepare themselves for their online classes including using online resources and their ideas, and seeking advice from other people who have more online teaching experience. The findings further indicate that EFL teachers in Bangladesh used institutional guidelines more often than Nepalese teachers. However, the practice of seeking advice from and collaborating with more experienced colleagues is more common in Nepal than in Bangladesh. For example, a teacher from rural Nepal mentioned that he could support his friends to use the video conferencing platform:

I could learn by attending courses through online mode delivered by the Nepal Open University. In that case, we could support the friends from TU. The other 4 teachers are taking classes in the master's program and I could train my other 4 friends and now they can use zoom comfortably. I help colleagues from Nepalgunj who would like to learn about using zoom.

The findings further reveal that their organisations provided some training to support teachers and students to cope with the challenges associated with online education practices. For instance, teachers from both countries, Nepal (*n* = 8) and Bangladesh (*n* = 14), reported that they received training from their organisations. Teachers also reported that they received training from specialised training institutions (*n* = 13) as well as from their colleagues (*n* = 10). Additionally, a handful of teachers from both countries managed to self-train through trial and error and YouTube tutorials (*n* = 5).

### Constraints and challenges in online teaching and learning

The findings indicate that EFL teachers face several challenges in running online classes. The major challenges include poor network, lack of digital skills, lack of technological support from institutions, low student attendance and motivation, lack of interaction, power cut, and difficulty in a demonstration, as indicated in the following extracts,

The challenges are manifold. First of all, the internet connection is a big issue. Then, the surrounding is not always classroom friendly… I have experience of students leaving the class just signing in with the email which means they were virtually present but in reality absent (Teacher A from Bangladesh).

One of the challenges is the unstable internet. Students have to keep their video and microphone off, so I can't see them all the time. Another challenge is ensuring interaction. Although some students participate actively by asking questions and sharing views, most students attend the class 'silently'. I can't always figure out if it is a connection issue or an attitudinal issue (Teacher B from Bangladesh).

Most teachers do not have technological knowledge… Let me give an example of my own college that more than 50 percent of teachers cannot correspond through email (Teacher A from Nepal).

The next one (challenge) is the use of technologies, technological devices. So, using these is also another challenge for both students and teachers (Teacher B from Nepal).

Teachers from Bangladesh also indicated some challenges in assessing students’ learning as the excerpt from an interview below reveals:

Students tend to plagiarise from different sources so I have to be always careful that… give them questions so that they cannot copy from the Internet still I think they somehow make a way through.

The teachers further reported that the students were passive in online classes, and sometimes it was very hard for them to make their students understand the content.

Similarly, students also reported that they faced several challenges in online classes. The major challenges for them included poor and unstable internet, power cuts, lack of patience, lack of devices to work on, inability to pay attention to the content because of getting distracted by the social media, unaccustomed to working in the online environment, expensive data packages and fear of online examinations.

I have to face the load shedding and the break of the internet. Also, I had to buy a laptop for the online class which, in this current economy, was a very difficult decision. Also, I cannot take notes properly as I cannot ask the teachers to repeat again and again. (Student A from Bangladesh)

Last time, when there was micro-teaching, one of my friends was in Bajang, and when he was doing his presentation, he was up the hill for a number of days because he could have a better network. (Student A from Nepal)

Indeed, the data indicated that most of the students from rural parts in the two countries had problems associated with internet connections. Teachers from both countries indicated that the cost of the internet was very high. Therefore, many students could not afford it: “*Some of them do not have enough money to view classes or to buy megabytes*.”

**Fig. 5:**
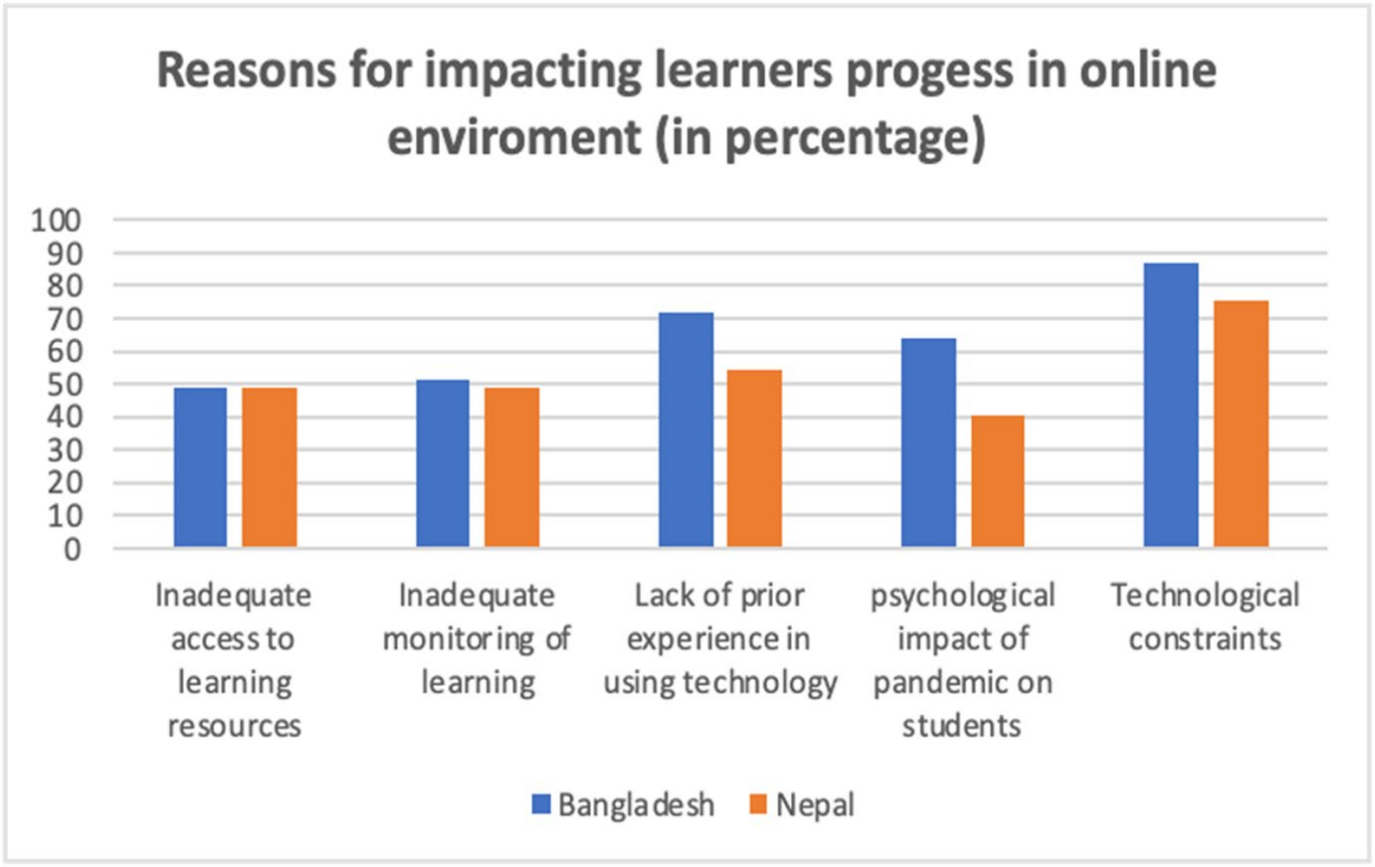
Reasons for impacting learners’ progress in online learning

Teachers from both countries indicated their concerns about their students’ learning progress and believed that several factors, as shown in the chart below, might have negative impacts on learning. Figure 5 summarises the survey responses.

As indicated by Fig. 5, teachers from both countries believe that technological constraints could be the biggest challenge or barrier to learners' progress in an online environment, which is followed by a lack of experience in using technology. Similar findings emerged through qualitative data. However, there were several indications that the EFL teachers and students received little or no support from their institutions, particularly in the context of Nepal. One of the teachers from a rural part of Nepal pointed out that there is no institutional policy in some educational institutions on the use of ICT, which impacts largely while running online classes.

The policy related to ICT made by the campus also does matter here. In some institutions, the laptops given by some donors are unused because nobody could use them. In some cases, the projectors donated by some institutions were on the cabinet.

It seems that teachers from both countries did not get enough training support from their institutions to manage online classes during the pandemic. However, the teachers from Bangladesh reported that their universities gave them access to more online journals during the pandemic. One of the Bangladeshi teachers further reported that students got technological support from their university: “*Based on the needs analysis my university authority is providing mobile smartphones to some of the students*.” Some students in Bangladesh also received data packages to use for their study.

### Mental wellbeing and preferences for face-to-face classes

Institutional support for mental wellbeing emerged as one of the pertinent concerns in this study. This means that the sudden shift to online education seems to have had a huge impact on students' and teachers' mental well-being. However, teachers and students received little support from their institutions. The data show that students, particularly from rural parts in Nepal, had anxiety associated with online education, and many Bangladeshi students felt depressed, as indicated by the following extracts:

I miss my friends. The anxiety was basically when there was a problem due to network failure or power cut. I would feel that then that I missed some lectures. I was not completely frustrated as I returned to Kathmandu soon. Had I been in the village, I could have been frustrated more (a student from Nepal).

… such support should be there because you know sometimes we feel depressed because of this online situation since we can’t meet everyone and go back to our traditional classes so our university should give us such mails just to encourage us (a student from Bangladesh).

Teachers’ mental well-being also seems to be affected during this pandemic. A few teachers indicated that the lack of a decent incentive affected their mental health. For instance, in the following excerpt, one of the teachers from Nepal points out the issue of salary and other incentives to be provided to the teachers during the Covid-19 crisis.

There’s a negative impact. Some young teachers who have joined campus with some enthusiasm have been demotivated. There was a feeling that more than being a teacher at the college level, the jobs at the government level seem better.

The participants suggested that support in the form of basic training in managing online resources, training to boost students’ morale during the pandemic, training for teachers on how to assist students psychologically, counselling classes, and workshops with experts in psychology could be useful to assist students and teachers psychologically. For instance, one of the participants contended, "*There could be a talk [Sic] session with the students to talk and discuss* their *situations so that they can be mentally prepared to embrace the online mode of learning."*

Both teachers and students further reported that they found online teaching too mechanical, and they did not feel comfortable with the classes as they had a feeling of not having control over their class. Furthermore, they indicated that there was a lack of sharing and a detachment from colleagues and friends and that they often found it very difficult to deliver content. This usually created anxiety among the teachers.

Similarly, many students from both countries indicated they found online classes too boring and monotonous, and they had limited opportunities for discussion in online classes. They felt like the classes were not natural. During the interviews, almost all the students reported they preferred face-to-face classes to online classes.

A few teachers also reported that their students were frustrated as they lacked technical skills*: “…some of the students are really frustrated because of these technical things that they’re not used to do it and suddenly they have to do so many things.”*

However, both the students and teachers pointed out some positive aspects of online classes such as, no need to commute, having more time with family, and more time for study and lesson planning. A few participants also reported that they were using this pandemic as an opportunity to be techno-friendly.

### Rules, roles, and practices

There are several indications that institutional or social norms/rules, division of activities, and hierarchy appeared as major issues during the transition from face-to-face to online education. For instance, a student in the following excerpt mentions how they are supposed to work (including their roles) during online classes.

In online classes, since we have to do all things by ourselves, if we can't do it, it becomes a burden to us. So our focus shifts from learning to how to solve that issue. In face-to-face, we can ask our friends and teachers but in online mode, since a teacher will be teaching us, it would be odd to disturb in the middle of the lecture.

The data also reveal a hierarchy that affects student participation in online classes. The following excerpt from a Bangladeshi student shows how hierarchy affected the participation of the learners.

Sometimes we have problems asking questions like we think that should we ask questions to the teachers, what he or she might think, or should we just shut …us down ...this makes problems.

However, the findings indicate students helped each other to face their challenges. For instance, one of the Nepalese students stated they supported their friends when they had difficulty joining online classes. Their roles became valuable to mitigate or solve the issues that appeared in online classes. During an interview, he mentioned,

They also share the problems in a group, such as the problems related to a network failure and so on. We also would tell them that we understand their problems.

Furthermore, one of the teachers from Nepal stated that different members of his university had performed different roles, and his university was committed to providing good support to its students and staff. For instance, the vice-chancellor of his university was leading himself to managing training for online classes. He further mentioned that they were connected with their students through Messenger to solve several issues, including emotional issues. He stated,

The VC is leading the team himself. The leadership is very much supportive.

Students do suffer from affective issues. But we try to keep them connected through Messenger. We even share study questions through Messenger. We try to keep them motivated.

This shows how different people at different levels are playing roles to cope with the challenges during the pandemic. For example, another teacher from Nepal mentioned they worked with other constituent campuses, and they had even developed a new pedagogical model that they wanted to adopt after the pandemic.

We have constituent campuses. We are using technology to connect with them. We have visualised a hybrid model of pedagogy after the pandemic. I do not think it will be 100% face-to-face or 100% online. It will be a hybrid model of teaching - a combination of face-to-face and online. So even though the situation is normal, we will continue a hybrid mode of pedagogy.

This points out the fact that teachers perceive blended learning, or a hybrid model, as a new norm in their post-Covid pedagogy.

## Discussions

Guided by the activity theory (Engeström, 1987, 1999, 2001), this study explores what and how digital tools have been used, how teachers and students of English/English education experience online learning, the impact on their mental wellbeing, and the constraints and challenges both teachers and students face during online learning in the higher education setting.

The subjects are teachers and students in higher education institutions in the activity systems who carry out object-oriented activity mediated by the use of digital artifacts. The object in the teachers' activity system is engaging students in online learning, and the object in the students' activity system is getting engaged in online learning during the pandemic. As seen in the first theme—mediating digital artifacts, the widely used digital tools in both countries are Facebook, Zoom, Google Meet, email, Messenger group and WhatsApp, and the digital devices are laptops and smartphones. One of the interesting findings is the context-sensitive use of a digital tool while transitioning to online teaching and learning. For instance, since the teachers in Nepal have no access to standard LMS in their local contexts, they seem to be creatively using Messenger group as their self-designed LMS, which corroborates the findings of Oberg and Daniels (2013) and Shah et al. (2020), who found that, in the absence of standard LMS, teachers use inexpensive and widely available tools to personalise instructions. What is surprising is that teachers identified the very distinct potentials of this social networking tool, Messenger group, which is primarily created for a chatting purpose. They set up their own e-rules, i.e., no participants can have informal communication in this Messenger group and established this as a platform to share the information and documents related to their academic activities. Thus, the self-created rules agreed by their own community to operate a digital tool seem to be helping them to carry out object-oriented activity. In line with Song and Fox (2008) who contend that digital tools provide novel, creative and entertaining learning contexts, the teachers could rightly identify the action potentials of the digital artifact they used.

Another unanticipated finding is that many teachers and students plan to use digital artifacts after the Covid-19 crisis contexts. The likely cause for this is the training they received during the pandemic, and their perceived usefulness and satisfaction affirming the findings of Vanitha and Alathur (2021) that the perceived usefulness and satisfaction of learners in higher education improved the adoption of digital technologies for teaching and learning. Their plan to use digital artifacts in the future may also be influenced by personal outcome expectations, as these types of expectations significantly influence learners' continuous intention to use a digital artifact—learning management system (Alzahrani & Seth, 2021). This finding implies that blended learning (a new normal) will get normalised in university settings in the near future.

It is equally intriguing that a large number of teachers from both Nepal and Bangladesh use online resources created by an online community and seek advice from the ones who have more online experience. They rely on each other and act as a community in a way that is comfortable for them during this transition.

The members of the community also play a very crucial role in helping each other and mitigate challenges that emerge while performing certain actions during teaching online. The primary challenges both teachers and learners face are: limited access to the network, power cut, issues related to learner engagement, possession of low-end devices and lack of competence to handle web tools and online resources which are in line with the findings of Khan et al. (2012) and Laudari and Maher (2019). The contradictions in activity systems emerge due to these challenges, such as there is an aggravated contradiction as teachers do not have access to a good network, yet they have to engage learners in teaching and learning. So the contradiction exists between two elements of the activity system, i.e., between mediating artifacts and objects. It is one of the secondary contradictions. Likewise, learners' engagement is not in line with teachers' anticipation even though teachers try to achieve this, in such a scenario, the contradiction is between teachers' and student's activity systems. Engeström (2001) argues the contradictions between two activity systems is a quaternary contradiction. He claims, "contradictions generate disturbances and conflicts, but also innovative attempts to change the activity" (p. 137). In this study, teachers searched alternatives and used technology as they saw fit in their local contexts, even in an unconventional manner (i.e., use of social platforms as LMS) which also corroborates with the findings of Shah et al. (2020). Lack of policy to guide the use of digital technologies also emerged as one of the challenges, which corroborates the findings of Mohammadi et al. (2021).

This study also unfolds learners' limited access to the appropriate devices as one of the challenges, which confirms Shrestha's (2016) findings. Despite having several challenges, teachers and learners share their difficulties and devise some transformative ways to mitigate these challenges to achieve their objectives. Shah et al. (2020) argue that the communication-collaboration-knowledge-building conception of ICT use is necessary for the effective implementation of ICT in higher education. The study shows that some teachers self-initiate specific actions, such as running training programs for their colleagues to use ICT to run online classes during this pandemic. This shows that societal and collaborative practices emerge due to the underlying tensions (Engeström, 1999), i.e., the lack of competence in handling mediating artifacts to run online classes in the activity system.

Amidst the challenges and uncertainties, the issues of mental wellbeing also prominently appear in this study. When subjects (teachers) interact with the institutional community but do not get appropriate support to perform the object-oriented activity, they show frustration. For example, the teachers in this study accentuate their doubt whether they would get a salary for teaching online during the pandemic or some incentives for the guest lectures that the teacher invited while he was having online classes.

The issue of mental wellbeing seems to be closely connected to the operation of digital artifacts. Due to poor network and power cut, learners could not join classes as expected, consequently, they were anxious and frustrated. The second-order barriers, as Laudari and Maher (2019) mention (such as teachers’ limited competence in using technology) posed a challenge for the teachers, and also the tension that emerges between mediating artifacts and the objects of the activity systems made them more psychologically feeble (Christakis & Christakis, 2020; Grubic et al., 2020) and socially isolated (Tereda, 2020). The finding of worsening mental wellbeing of the learners resonates with the finding by Cao and their colleagues (Cao et al., 2020), who report that 25 percent of Chinese students experienced anxiety during the pandemic. The study also reveals that basic training, support and counselling in managing online classes can be useful in dealing with the issues of learners’ mental wellbeing during a crisis.

It is also stimulating to observe how netiquette is integrated into the hierarchical relationship that exists between teachers and students in activity systems. The students mention that they do not want to disturb their lecturers who would be delivering a class, as a result, the students remained passive. Therefore, learners’ perceived rules and the vertical relationship between the learners and teachers play some roles to affect the level of interaction between them. The division of labour between the community members does affect online teaching and learning.

## Conclusion

This study explored students’ and teachers’ experiences of getting involved in online classes during the pandemic. The findings demonstrate that students and teachers mostly use laptops and smartphones, and digital tools, such as Facebook, Zoom, Google Meet, email, Messenger group and WhatsApp. They seem to adapt the potentials of the artifacts to their own local context and use them in the best possible way rather than merely perceiving the overt potential designed by the designers of the artifacts. As regards the community of practice, they create a strong relationship to help each other regulate their online teaching and learning, for instance, teachers seek help from experienced ones.

The teachers' and students' challenges can be categorized into school-level barriers (first-order barriers), teacher-level barriers (second-order barriers) and system-level barriers. The challenges such as lack of ICT infrastructure, poor Internet connection fall under first-order barriers; lack of efficacy and confidence of teachers in handling web tools fall under second-order barriers, and the system level barriers comprise lack of institutional policy and lack of clarity in the assessment which this study revealed. Poor internet connection, frequent power cut, and lack of ICT competence appear as the major challenges. The study also indicates mental wellbeing issues both with students and teachers.

The findings of the study have some important implications for the policymakers who frame a policy for online education. It is also equally useful for higher education institutions to understand their roles to support e-pedagogy and manage any educational crisis. It can be largely beneficial for teachers to identify the constraints and challenges of online teaching and learning during crises and devise appropriate strategies beforehand to address such challenges. It is a guiding study for students of higher education as it has the potential to make them understand their roles in an online learning environment. As this study indicates the significance of social learning platforms in teaching and learning, software designers may consider either including social learning spaces or embedding external social learning platforms in educational software that they design. On top of that, the empirical documentation of the educational practices made in both developing countries in this never-to-expect crisis itself has a huge value as different types of crises, such as the ones caused due to political instability, natural calamities, epidemic and the like which seem quite frequent in these parts of the world, can impact educational activities anytime in the future.

The major limitation of this research is its scope, as it was limited to the teachers and students of English and English education in higher education institutions. Therefore, it can be suggested that teachers and students of English and English education might be more digitally competent than the teachers and students of other subjects who are in higher education or vice versa. Nonetheless, it has presented only indicative results limiting its generalizability to a wider population. Being limited to only two developing countries, Nepal and Bangladesh, this study may not be taken as a strong case to understand the practices of online education during the pandemic in all developing countries. Moreover, this study has not explored learners' equitable access to technology and their issues in relation to the digital divide during crisis contexts, which are potential areas for future research.

## Data Availability

Not applicable
